# Advancements in the Understanding of the Genetics of Obsessive-Compulsive Disorder (OCD)

**DOI:** 10.1007/s11920-026-01705-0

**Published:** 2026-07-31

**Authors:** A. Christelle Doppenberg, Carolina Cappi, Nora I. Strom, James J. Crowley, Dale R. Nyholt, Brittany L. Mitchell, Eske M. Derks

**Affiliations:** 1 Brain and Mental Health Program, QIMR Berghofer Medical Research Institute, Brisbane, QLD Australia; 2 School of Biomedical Sciences, Queensland University of Technology, Brisbane, QLD Australia; 3 Department of Psychiatry, Robert Wood Johnson Medical School, the State University of New Jersey, Rutgers, Piscataway, NJ USA; 4 Department of Psychiatry, Psychosomatics and Psychotherapy, Center of Mental Health, University Hospital Würzburg, Würzburg, Germany; 5 Department of Psychiatric Phenomics and Genomics (IPPG), Ludwig- Maximilians University Munich, Munich, Germany; 6 Department of Genetics, University of North Carolina at Chapel Hill, Chapel Hill, NC USA

**Keywords:** Obsessive-Compulsive Disorder, Heritability, Polygenic, GWAS, Rare Variants, Genetics

## Abstract

**Purpose of Review:**

This review summarizes recent advances in the genetics of Obsessive-Compulsive Disorder (OCD), their contribution to understanding disorder biology, and implication for clinical translation.

**Recent Findings:**

Recent GWAS identified 30 genome-wide significant loci and prioritized 25 putatively causal genes. Rare variant studies implicated specific genes, including *CHD8*, *CELSR3*, *SLITRK5*, and *QRICH1*. Evidence from common and rare variants support brain- and immune-related pathways. Genetic overlap with obsessive compulsive symptoms and other psychiatric disorders indicate shared underlying biology. Current evidence is largely based on individuals of European ancestry, although global efforts are underway to improve ancestral diversity in OCD genetics. Given the urgent need for improved treatment, genetically informed clinical translation approaches hold promise, including pharmacogenetics and drug repurposing.

**Summary:**

Recent advances in OCD genetics support a highly polygenic architecture, implicate specific neuro-biological and immune pathways, and provide new opportunities for clinical translation.

## Introduction

Obsessive-Compulsive Disorder (OCD) is a chronic and debilitating psychiatric disorder that affects approximately 1% of the population[1], characterized by recurrent and intrusive thoughts, impulses, or images (obsessions), and repetitive behaviors that are usually performed in response (compulsions)[2]. Individuals with OCD can experience a variety of symptoms that differ widely between people and may fluctuate in presentation and severity throughout their lives[2]. People with OCD also face significant clinical challenges, with an average diagnostic delay of 7 years in young people today[3], and well over 10 years in older adults[4]. Moreover, nearly half of patients fail to respond to first-line treatments[5–7]. These factors contribute to an elevated risk of suicide and increased overall mortality[8–10].

There is strong evidence for a genetic contribution to OCD, with twin-based heritability estimates reaching 47% [11–14]. Recent large-scale genomic studies have identified common and rare variants implicated in OCD, with rare variants making a substantial contribution to disease risk, accounting for ~ 10.4% of OCD heritability[15–23]. Exploring the genetic contribution to OCD risk may help identify biological processes involved, contributing to a better understanding of the disorder and informing the development of more effective treatments. The aim of this review is to outline recent advances in the genetics of OCD and their implications for clinical translation.

### Current Insights Into OCD Based on Common Genetic Variants

#### Genetic Risk of OCD and Heritability

The genetic risk of OCD is increasingly being characterized through Genome-Wide Association Studies (GWAS, Glossary), which have identified multiple loci (Glossary) associated with disorder risk[22,24–26] (Table 1). The largest and most recent GWAS reported the first significant findings for OCD, identifying 30 independent loci[22]. These results support a highly polygenic architecture in which many common variants contribute small effects to disorder risk[22]. In this study, SNP-based heritability (Glossary) was estimated at 6.7% on the liability scale, lower than some previous SNP-based estimates[21,24,27]. This reduction likely reflects heterogeneity in phenotype definition, ascertainment strategies, and sample composition across cohorts. Heritability estimates were higher in clinically ascertained and comorbid subgroups, reaching 16.4% and 13.3%, respectively[22]. Polygenic Scores (PGS, Glossary) derived from this GWAS were significantly higher in cases than in controls (*b* = 0.41, *95%CI*[0.35–0.47], *p* = 1 × 10^− 16^)[28].

**Fig. 1:**
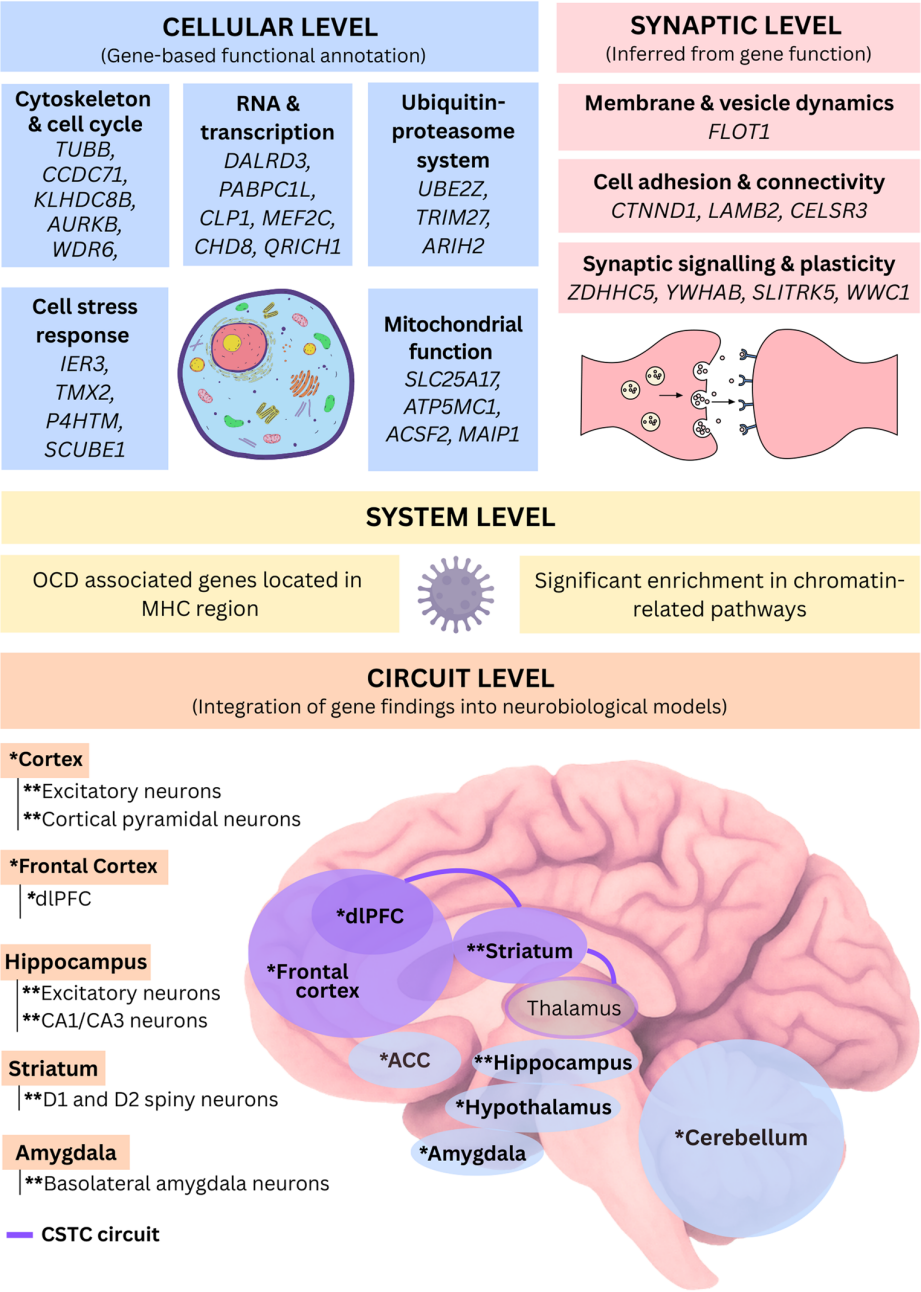
Current Genetic Evidence Informing the Neurobiology of OCD Based on Common and Rare Genetic Variants Note. This figure presents a hypothesis-driven synthesis inferred from genes prioritized for OCD. * = significant tissue enrichment; ** = significant cell-type enrichment. ACC, anterior cingulate cortex; CSTC, cortico-striato-thalamo-cortical; dlPFC, dorsolateral prefrontal cortex; MHC, Major Histocompatibility Complex

GWAS have identified loci associated with OCD, yet translating these associations into putatively causal genes is complicated by the fact that the majority of associated SNPs fall in non-coding or intergenic regions. To address this, complementary gene-prioritization strategies can be applied. Positional mapping assigns variants to nearby genes based on genomic location, whereas functional mapping integrates biological annotations, such as gene expression data, to prioritize genes whose regulation may be influenced by associated variants. In the recent OCD GWAS, 251 genes were implicated by at least one of five gene-mapping approaches, and 48 genes were implicated by at least two approaches. Among these, 25 genes were further prioritized as putatively causal using a colocalization-based method. Evaluating these prioritized genes in relation to biological pathways and processes provides insight into the molecular and cellular mechanisms underlying OCD (Fig. 1).

**Table 1:** Summary of major genetic studies in OCD based on common and rare genetic variants

Study	Method	Trait	*N*_Cases_	*N*_Total_
Jung (2026)[20]	WGS study	OCD (case/control)	2,561	15,535
Abdallah (2025)*[15]	CNV study	OCD (case/control)	549	3,411
Halvorsen (2025)[18]	CNV study	OCD (case/control)	2,248	5,856
Jung (2025)[19]	WES study	OCD (case/control)	6,071	44,089
Strom, Gerring, Galimberti & Yu (2025)[22]	GWAS	OCD (case/control)	53,660	2,098,077
Wang (2025)*[29]	WES study	OCD/CTD (case/control)	8,165	11,861
Strom (2024)[30]	GWAS	OC symptoms (quantitative)	-	33,943
Mahjani (2022)[23]	CNV study	OCD (case/control)	993	2,242
Bralten (2021)[31]	GWAS	OC symptoms (quantitative)	-	650
Burton (2021)[32]	GWAS	OC symptoms (quantitative)	-	5,018
Halvorsen (2021)[33]	WES study	OCD (case/control)	1,263	12,843
Cappi (2020)*[16]	WES study	OCD (case/control)	184*	961*
Smit (2020)	GWAS	OC symptoms (quantitative)	-	8,267
IOCDF-GC (2018)	GWAS	OCD (case/control)	2,688	9,725
Den Braber (2016)	GWAS	OC symptoms (quantitative)	-	6,931
Gazzellone (2016)	CNV study	OCD (case/control)	307	4,186
Mattheisen (2014)	GWAS	OCD (case/control)	1,406	2,904
McGrath (2014)	CNV study	OCD/TS (case/control)	2,699	4,488
Stewart (2012)	GWAS	OCD (case/control)	1,465	7,022

Note: Studies marked with “*” included parent-child trios. *CNV =* copy number variant, *CTD* = Chronic Tic Disorder, *GWAS =* genome-wide association study, *OCD* = obsessive-compulsive disorder, *OCS* = obsessive-compulsive symptoms, *WES* = whole-exome sequencing, *WGS* = whole-genome sequencing

#### Evidence Implicating Brain-Related Processes in OCD

Several risk genes were mapped to brain-related processes, including neurodevelopmental and synaptic mechanisms (Fig. 1). Notably, *CTNND1*, prioritized across three gene-based tests and identified as putatively causal, showed strong colocalization with expression signals in the dorsolateral Prefrontal Cortex (dlPFC). This region has been systematically implicated in the neurocircuitry of OCD[34,35]. In addition, the two genes with strongest converging evidence for causality, *WDR6* and *DALRD3*, are involved in neuronal and synaptic processes[36,37]. On the same genomic locus, *CELSR3* was associated with OCD, and has previously been linked to Tourette Syndrome, which is closely related to OCD clinically and genetically, and recognized in the DSM-5 through the “tic-related” specifier for OCD[2,38,39]. *CELSR3* was also implicated in rare variant studies of OCD (Sect.  Current insights into OCD based on rare genetic variants)[19].

Established neurobiological models of OCD emphasize dysfunction in distributed brain circuits. The Cortico-Striato-Thalamo-Cortical (CSTC) circuit, comprising primarily the prefrontal cortex, striatum, and thalamus, has long served as the dominant framework for understanding OCD pathophysiology[34,35]. Enrichment analyses from the recent OCD GWAS further support CSTC circuitry in OCD pathophysiology[22]. Significant enrichment was observed in the frontal cortex, as well as in dopamine D1 and D2 receptor-expressing Medium Spiny Neurons (MSNs) within the striatum. Notably, rare variant studies of OCD also implicated these neuronal populations within the striatum (Sect.  Current insights into OCD based on rare genetic variants). Dopaminergic signalling within the striatum has long been associated with OCD as part of CSTC circuitry (Fig. 1)[40].

Accumulating evidence pointing to other networks in OCD, such as frontolimbic, frontoparietal, and cerebellar systems, suggest broader circuit-level dysfunction beyond the classical CSTC model[35,41]. Based on the recent OCD GWAS, significant tissue enrichment of the genetic signal associated with OCD was observed in cortical (Anterior Cingulate Cortex, Cortex) and subcortical regions (Amygdala and Hypothalamus)[22]. Notably, significant enrichment of excitatory neurons was found in the cerebral cortex and the hippocampus (Fig. 1)[22]. Consistent with this, two genes prioritized as putatively causal in the recent OCD GWAS, *MEF2C* and *ZDHHC5*, contribute to excitatory neuronal function[42–44]. Evidence from mouse models suggests that loss of *ZDHHC5* reduces excitatory synapse density in the hippocampus[43]. These findings are consistent with glutamatergic pathways in OCD, consistent with prior OCD GWAS prioritizing glutaminergic genes[24]. Disruption of excitatory-inhibitory balance has been implicated in the pathophysiology of OCD[45].

These genetic findings are complemented by additional evidence from mouse models, Notably, *SLITRK5*-knockout, *HOXB8*-null mice and mice with a genetic deletion of *SAPAP3* (*DLGAP3*) exhibited increased anxiety and compulsive grooming behaviour, mirroring OCD features[46–50]. These behaviors were alleviated by Selective Serotonin Reuptake Inhibitors (SSRIs), which are commonly used in the treatment of OCD. Moreover, *SLITRK5-* knockout mice showed selective overactivation of the orbito-frontal cortex, striatal anatomical abnormalities, and alterations in glutamate receptor composition, consistent with prior evidence regarding OCD neurobiology[48]. These findings may be relevant to humans. *SLITRK5* and *HOXB8* have been implicated in rare variants studies of Tourette Syndrome and Trichotillomania respectively (Sect.  Current insights into OCD based on rare genetic variants)[46, 51]. Both disorders are related to OCD genetically and classified within the Obsessive-Compulsive and Related Disorders (OCRD) diagnostic group[2]. Overall, although not all genes implicated in mouse models have been replicated in humans, they map on pathways consistent with established OCD neurobiology, including synaptic function, glutaminergic signaling, and significant enrichment in key brain regions.

#### Evidence Implicating Immune-Related Processes in OCD

Beyond genes implicated in brain-related processes, several risk genes for OCD map to the Major Histocompatibility Complex (MHC) locus, a region that is central to adaptive immune function[22]. A link between OCD and the immune function has been documented previously[52]. For example, many immune-related conditions sometimes present with obsessive-compulsive symptoms[53]. These include Pediatric Autoimmune Neuropsychiatric Disorders Associated with treptococcal infections, and Pediatric Acute-onset Neuropsychiatric Syndrome[52]. Consistent with this, significant genetic correlations were observed between OCD and several autoimmune disorders[22]. Notably, some of these associations were negative, specifically with Crohn’s Disease, Ulcerative Colitis, and Inflammatory Bowel Disease[22]. Family-based studies also supported this pattern, reporting a negative genetic correlation between OCD and Type 1 Diabetes[54]. However, an overall risk of autoimmune disorders was increased among relatives of individuals with OCD. This contrasts with GWAS-based findings[54], suggesting unresolved heterogeneity in this relationship. Current evidence suggests that immune involvement in OCD may characterize a specific subtype, rather than reflecting a unifying mechanism across the disorder as a whole[52].

The prioritization of MHC genes is not unique to OCD and has been observed in other psychiatric disorders including Autism Spectrum Disorder (ASD), Attention-Deficit/Hyperactivity Disorder (ADHD), and Anxiety Disorders[55–57]. However, the pattern of negative correlations is somewhat unusual compared with other psychiatric-immune relationships. Within the MHC locus, *TRIM27* was prioritized as a putatively causal gene for OCD[22]. This gene has been associated with autoimmune diseases, particularly psoriasis and Crohn’s disease, the latter showing a negative genetic correlation with OCD[58]. In addition, *QRICH1*, an MHC gene associated with OCD in the recent GWAS, was also implicated in rare variant studies of OCD (Sect.  Current insights into OCD based on rare genetic variants) (Fig. 1). Mouse models have raised evidence for immune processes taking place in the brain that may contribute to OCD-like behavior[42,49]. These processes are thought to involve microglia, the resident immune cells of the brain. In these models, disruptions in microglial function of the *HOXB8* and *MEF2C* genes induced OCD-like and repetitive behaviors, respectively[42,49]. Although the latter was originally studied in the context of ASD, *MEF2C* was implicated in the recent OCD GWAS[22].

Extending gene-level findings further, pathway analyses conducted on the 251 genes prioritized for OCD revealed significant enrichment in chromatin structure-related pathways[59]. Chromatin remodeling regulates several processes including neuronal differentiation, synaptic gene expression, and immune signaling. Therefore, disruptions may alter neurotransmission and immune function, potentially contributing to OCD symptoms and their variability in response to stress or infection. Evidence from rare variants also supports the involvement of chromatin-related processes in OCD (Sect.  Current insights into OCD based on rare genetic variants).

#### Insights from Obsessive-Compulsive Symptoms

While ~ 1% of individuals meet criteria for a clinical diagnosis of OCD, a substantially larger proportion of the population experiences subclinical Obsessive-Compulsive Symptoms (OCS). These are thought to lie on a continuum, with clinically diagnosed OCD representing the extreme end. Therefore, investigating the genetic architecture of OCS provides an opportunity to study larger and more representative population samples, and to assess the extent to which OCS and OCD share underlying biological mechanisms. Several GWAS have been conducted for OCS[30,32,60–62] (Table 1). SNP-based heritability estimates for OCS (~ 4.1%) are lower than for OCD (~ 6.7%)[23, 30], which is typically observed for non-clinical phenotypes[63]. Symptom-level GWAS have identified loci that have been mapped to specific risk genes[32,61] (Table 1). Of particular interest, *PTPRD*[32] was also implicated in prior OCD GWAS[22,25], and *RFXANK*[61] is located within the MHC region. The latter suggests that immune processes may extend beyond immune-specific subtypes of OCD, which are thought to represent often more severe cases. However, the most recent and well-powered GWAS of OCS did not identify immune-related findings[30].

In genetic correlation analyses, OCS showed the strongest genetic association with OCD among all tested traits[30]. Both displayed highly similar patterns of genetic correlation with other psychiatric disorders, suggesting that OCS may capture a large proportion of OCD genetic risk[22,30]. Notably, this overlap appears primarily driven by compulsivity, which shows significant genetic correlation with OCD and higher SNP-based heritability than obsessionality (0.116 vs. 0.058)[64], suggesting partially distinct genetic architectures. Estimates from twin models indicate that both dimensions are significantly heritable, with modestly lower heritability observed for obsessionality compared to compulsivity[65,66].

### Genetic Overlap between OCD and Related Traits

OCD is often comorbid with other psychiatric disorders, with an estimated 65–85% of individuals with OCD experiencing at least one co-occurring psychiatric condition during their lifetime[22]. This clinical overlap may, in part, reflect shared genetic risk across psychiatric disorders. Consistent with this, genetic correlation analyses indicated significant associations between OCD and all tested psychiatric disorders, with the strongest correlations observed for Anxiety Disorders (rg = 0.7), Major Depressive Disorder (MDD) (rg = 0.55), and Anorexia Nervosa (AN) (rg = 0.52)^22^. A cross-disorder GWAS of OCD and AN reported a significant genetic correlation (rg = 0.49) and cross-disorder SNP-based heritability (h^2^ = 0.21), although no significant loci were identified[67]. The combined AN/OCD phenotype exhibited positive genetic correlations with other psychiatric disorders, as well as negative correlations with metabolic phenotypes, including BMI and triglycerides[67]. In addition, differential genetic risk for OCD was observed between comorbid and non-comorbid OCD. Polygenic risk scores for OCD showed the strongest predictive performance in OCD occurring alone, and weakest with co-occurring MDD[68]. Beyond pairwise correlations, in genomic structural equation modeling of 14 psychiatric traits, OCD loaded on a shared compulsivity factor, alongside Anorexia Nervosa, Tourette Syndrome and Anxiety Disorders[69].

### Current Insights into OCD Based on Rare Genetic Variants

While common variants account for most of the inherited liability to OCD, rare variants, particularly *de novo* (Glossary) variants, can also make substantial contributions to disease risk[21]. Although they occur at low frequency in the population, *de novo* variants can have large individual effects and have been instrumental in systematic risk-gene discovery for related neurodevelopmental disorders, including ASD, Schizophrenia, and developmental delay[70]. It is now well established that both common and rare variants contribute to the genetic architecture of psychiatric disorders along an “allelic spectrum,” with their relative contributions varying by diagnosis and providing complementary rather than mutually exclusive lines of evidence[21,70]. Three major classes of rare variation have been investigated in OCD: Copy Number Variants (CNVs, Glossary), rare sequence variants identified through Whole-Exome Sequencing (WES, Glossary), and, more recently, rare regulatory non-coding variants identified through Whole-Genome Sequencing (WGS, Glossary) (Table 1).

The first systematic genome-wide CNV study of OCD jointly analyzed 1,613 OCD cases and 1,086 Tourette Syndrome cases, reporting an elevated rate of neurodevelopmental deletions and identifying 16p13.11 as a recurrent locus of interest[71]. Subsequent studies confirmed enrichment of rare CNVs at loci also implicated in ASD and Schizophrenia, including 16p13.11, 22q11.2, and 16p11.2[72]. A population-based analysis of the Swedish EGOS cohort identified potentially damaging CNVs in approximately 9% of OCD probands and 8% of Chronic Tic Disorder (CTD) probands. CTD is related to OCD clinically and genetically, and is recognized in the DSM-5 through the “tic-related” specifier for OCD[2]. These variants were significantly enriched in CTD cases with co-occurring ASD and were associated with specific OCD symptom dimensions, particularly Ordering and Checking[23]. The largest case-control CNV study of OCD to date, conducted in the Scandinavian NORDiC cohort, demonstrated a significant excess burden of rare CNVs in OCD cases (OR = 1.12), particularly large (> 1 Mb), ultra-rare CNVs overlapping loss-of-function-intolerant genes[18]. Importantly, OCD cases carrying deleterious CNVs had lower polygenic scores (Glossary) for psychiatric conditions, suggesting that rare large-effect structural variants and common polygenic risk may represent partially independent contributors to OCD susceptibility. Complementing these array-based studies, a recent analysis applying CNV detection to WES data from pediatric OCD trios identified more than 11-fold enrichment of rare de novo CNVs in probands compared with controls[15], providing further evidence for the contribution of structural variation to OCD risk and demonstrating the value of integrating multiple variant-detection strategies.

The first systematic WES study of OCD analyzed 184 parent-proband trios and demonstrated a significant excess of de novo damaging variants in cases, identifying *CHD8*, a chromatin remodeler also implicated in ASD, and *SCUBE1* as high-confidence risk genes[16]. Pathway and network analyses in this study highlighted enrichment in immune-related pathways, particularly the complement system, as well as sodium ion homeostasis and neurotrophin/tyrosine kinase signaling. These findings are consistent with prior reports of elevated pro-inflammatory markers in pediatric OCD[52] and further support immune system involvement in OCD pathophysiology described above. A subsequent larger WES analysis combining trios, quartets, and singleton cases (1,313 cases vs. 11,580 controls) replicated the excess burden of de novo damaging variants and rare loss-of-function variants in loss-of-function-intolerant genes, with effects particularly pronounced in male probands[33]. This study identified *SLITRK5* as the most significant single-gene association in case-control analyses (OR = 8.8), a gene previously implicated in OCD-like behaviors in knockout mouse models, thereby providing supportive evidence across human genetic and animal model studies.

Two recent large-scale WES studies have further expanded this catalog. Wang et al. (2025) jointly analyzed 3,964 individuals with OCD, Chronic Tic Disorder (CTD), or both, identifying 36 high-confidence risk genes with very large estimated effects (mean odds ratio ≈ 57)[29]. These effect sizes reflect conditional effects derived from specific mutations in a subset of OCD patients with comorbid CTD. Of note, this distinguishes them from GWAS-style burden ORs and should be emphasized to avoid misinterpretation as broadly generalizable risk estimates. In parallel, a WES analysis of 6,071 OCD cases identified *CHD8* as genome-wide significant for OCD, with *CHD8* and *CELSR3* both reaching genome-wide significance in a meta-analysis combining OCD and CTD[19]. Notably, these two studies used overlapping samples and similar Bayesian statistical frameworks, yet reached partially divergent gene-level conclusions. These differences likely reflect analytic choices related to the inclusion of singleton case-control data, variant-class thresholds, and prior assumptions. Both studies are currently preprints. Nonetheless, the recurrent identification of *CHD8*, *CELSR3*, *WWC1*, and *QRICH1* across independent analyses provides converging evidence for these genes as bona fide OCD risk genes.

Beyond gene-level findings, recent rare-variant studies have begun to delineate the biological pathways and mechanisms implicated in OCD. Network propagation and Gene Ontology analyses implicated specific neurodevelopmental processes, including axonogenesis, microtubule-based movement, ciliary biology, cell polarity, and Hippo signaling pathways that have also emerged in studies of ASD and other neurodevelopmental disorders[29]. Jung et al. (2025) further demonstrated that genes regulated by *CHD8* are enriched for both rare variants and common variants from OCD GWAS, establishing a direct link between rare and common variant risk through shared regulatory pathways[19] (Sect.  Current insights into OCD based on common genetic variants). Chromatin organization is consistently emerging as a central biological theme in OCD genetics across both rare- and common-variant studies, and its disruption may alter neuronal development as well as immune responses and neuroimmune interactions. These findings are supported by evidence from the broader OCD genetic literature implicating immune-related pathways and the MHC region[22] (Sect.  Current insights into OCD based on common genetic variants), as well as with earlier WES findings of enrichment for complement and immune signaling pathways[16].

A striking feature of these rare-variant findings is their extensive pleiotropy across neurodevelopmental and psychiatric disorders. OCD/CTD risk genes show significant overlap with risk genes for ASD, developmental delay, and Schizophrenia, but not with congenital heart disease[29]. In addition, 33–36% of high-confidence ASD risk genes show evidence of association with OCD[19]. This pattern aligns with the substantial overlap observed at the level of common variants[22] (Sect.  Current insights into OCD based on common genetic variants) and supports a partially shared neurodevelopmental genetic basis across these conditions.

At the cellular level, rare-variant risk genes are enriched for expression in telencephalic projecting excitatory neurons, including cortical pyramidal neurons, hippocampal CA1/CA3 neurons, and basolateral amygdala neurons, and in striatal medium spiny neurons, including both D1 + and D2 + subtypes [29]. Notably, these cell-types directly overlap with those identified through common-variant analyses[22], providing evidence that rare and common genetic risk for OCD may act on shared neuronal substrates within CSTC circuitry (Sect.  Current insights into OCD based on common genetic variants). Spatiotemporally, OCD/CTD risk genes show elevated expression across both prenatal and postnatal brain development, with the strongest postnatal enrichment in the cerebellum and striatum, a developmental profile distinct from ASD and consistent with the typically later age of onset of OCD.

Phenotypic heterogeneity is emerging as a key consideration in rare-variant studies of OCD. OCD with co-occurring tics and OCD without tics show distinguishable, though overlapping, genetic architectures[29], and individuals with treatment-resistant OCD who are eligible for deep brain stimulation appear to be particularly enriched for large-effect rare variants[73]. Most recently, a WGS analysis of 2,561 OCD cases identified significant enrichment of rare, conserved variants in the *KNCN/MKNK1-AS1* antisense long non-coding RNA-protein-coding overlap region, with strong striatal co-expression of the implicated transcripts[20]. Although based on a single locus, this finding highlights an underexplored dimension of OCD genetics and suggests that non-coding regulatory variation may represent both a novel disease mechanism and a potential source of therapeutic targets.

### Ancestral Transferability of Genetic Findings in OCD

All GWAS of OCD and OCS to date have been conducted exclusively in individuals of European-ancestry, raising concerns about their transferability to other populations. The cross-ancestry performance of OCD Polygenic Scores (PGS) in predicting OCD risk has been investigated in both Indian[74] and East Asian samples[75]. In the Indian sample, OCD PGS significantly predicted OCD with modest effect sizes (mean odds ratio ≈ 2), while results in the East Asian sample were non-significant. This difference is likely attributable to better-powered GWAS used to construct PGS in the Indian sample[68], compared to the East Asian[24]. The OCD GWAS used in the East Asian study also showed limited PGS predictive performance within European samples, with comparable effect sizes (odds ratio ≈ 1.2)[76]. OCD PGS may demonstrate cross-ancestry performance, although further investigation is required. The recent OCD GWAS[22] now provides an opportunity to revisit PGS transferability across ancestral groups.

### Opportunities and Challenges for Clinical Translation

Given that approximately 50% of OCD patients do not show adequate response to first-line serotonergic medications, there is an urgent need to identify individuals at risk of reduced treatment efficacy and to better characterize treatment trajectories. While polygenic scores for OCD are continuing to improve, their current accuracy is not yet sufficient to reliably inform individual-level risk stratification (b ≈ 0.40, reflecting a ~ 0.4 standard deviation higher score in cases than controls)[28], and current GWAS explain only a small proportion of OCD heritability.

Only a few studies have examined whether treatment outcome is associated with genetic variants. OCD patients carrying deletions in neurodevelopmental disorder genes had significantly poorer response to multimodal treatment (Cognitive Behavioral Therapy and/or SSRIs), with carriers improving 16% on the Yale-Brown Obsessive Compulsive Scale compared to 47% in non-carriers[18]. Supporting evidence from a GWAS of treatment response in OCD (*N* = 804) suggested enrichment of genes implicated in glutaminergic and serotonergic neurotransmission systems[77]. PGS derived from the recent OCD GWAS were used to predict response to Cognitive Behavior Therapy in individuals diagnosed with OCD. However, the results showed no significant association[28]. Pharmacogenetics has emerged as a promising approach for improving precision medicine by identifying genetic factors associated with treatment response. Some evidence has been promising regarding MDD[78], yet evidence for OCD remains limited. While some encouraging findings have been reported for *CYP2D6* and *CYP2C19*[79], stronger effect sizes are likely to emerge from approaches that incorporate a broad range of genetic variants rather than focusing on single metabolizer genes[80]. While these opportunities are promising, polygenic scores and pharmacogenetic approaches currently remain primarily research tools and are not yet clinically actionable for OCD.

Advances in the prioritization of causal genes associated with OCD open new opportunities for drug repurposing, as applied previously for other neuropsychiatric conditions[81]. By identifying therapeutic applications for already-approved drugs, this approach circumvents the lengthy, costly process of developing novel therapeutics. There is emerging evidence supporting drug repurposing in OCD. For example, memantine, originally developed for Alzheimer’s disease, has been introduced as a treatment for OCD based on evidence implicating glutamatergic dysfunction[82]. To date, drug repurposing strategies in OCD have not been guided by genetic findings, although such approaches are cost and time efficient[83]. Putatively causal genes identified in recent OCD GWAS may represent potential targets for therapeutic development.

Several limitations should be considered when interpreting the current genetic evidence for OCD. Most loci identified through GWAS have small effect sizes and explain only a modest proportion of OCD heritability. Heterogeneity across cohorts may also contribute to variability in these findings. Rare variant studies generally involve smaller sample sizes and more clinically severe subgroups. Furthermore, putatively causal genes identified through statistical prioritization approaches do not represent definitive causal mechanisms. While the recent GWAS of OCD represents a major advancement, continued progress will require larger and more ancestrally diverse samples, as well as the integration of both common and rare variants. These developments are necessary to further elucidate the genetic architecture of OCD and facilitate the discovery of causal genes. Future work may also benefit from incorporating gene-environment (G×E) and gene-gene (G×G) interactions, as well as epigenetic mechanisms, to better capture the complex and context-dependent architecture of OCD risk. In addition, investigating a rather multidimensional perspective of OCD, including sex differences, X-chromosome variation, its broader context within the OCRDs, as well as specific symptoms, dimensions, and subtypes, is necessary to better capture the complexity and heterogeneity of the disorder. International consortia such as the OCD/TS working group of the Psychiatric Genetics Consortium (PGC) are working towards many of these goals[84]. Major initiatives are underway to address the under-representation of diverse populations in genomic OCD research. These include the Latin American Trans-ancestry INitiative for OCD Genomics (LATINO)[85], the Black EquaLity in OCD NeuroGenomics (BELONG) (Williams, 2026), as well as the Ancestral Populations Network (APN)[86], aiming to expand representation across Asian, Hispanic, and African populations.

## Key References

- Strom NI, Gerring ZF, Galimberti M, et al. Genome-wide analyses identify 30 loci associated with obsessive–compulsive disorder. Nat Genet. 2025;57(6):1389-1401.
  - This study represents the largest genome-wide association study of OCD to date, identifying 30 genome-wide significant loci and mapping 249 genes, including 25 putatively causal genes.
- Jung S, Caballero M, Smout S, Mahjani B. Rare Variants in Antisense Long Noncoding RNA–Protein-Coding Gene Overlap Regions Contribute to Obsessive-Compulsive Disorder. Biol Psychiatry Glob Open Sci. 2026;6(2):100683.
  - This study represents the largest whole-genome sequencing study of OCD to date and identified an association between OCD and rare variants in antisense long noncoding RNA-protein-coding gene overlap regions.
- Halvorsen MW, De Schipper E, Bäckman J, et al. A burden of rare copy number variants in obsessive-compulsive disorder. Mol Psychiatry. 2025;30(4):1510-1517.
  - This study represents the largest copy number variant study of OCD to date and found an increased burden of rare copy number variants, particularly large deletions and duplications affecting genes intolerant to loss-of-function variation.
- Jung S, Halvorson MW, Pedersen N, et al. Rare coding variation in OCD implicates shared genes with other psychiatric disorders. medRxiv. Published online December 23, 2025:2025.12.22.25342827. doi:10.64898/2025.12.22.25342827
  - This study represents the largest whole-exome sequencing study of OCD to date and found that rare coding variants are enriched in genes shared across multiple psychiatric disorders, supporting a shared genetic architecture. This study is currently a preprint.

## Data Availability

No datasets were generated or analysed during the current study.

## Glossary

Copy Number Variants (CNVs): Structural genetic variations involving deletions or duplications of segments of DNA.
De Novo variants: Variants that arise spontaneously in an individual through a mutation occurring in a parental germ cell or during early embryonic development,
Genome-Wide Association Studies (GWAS): Method based on common genetic variants (SNPs) used to identify statistically significant associations between genomic loci and a trait or disease within a population.
Heritability: Measure of how much of the variation in a trait is due to genetics.
Loci: A locus (plural: loci) is a specific, fixed position on a chromosome where a particular gene or genetic variant is located.
Pleiotropy: Phenomenon in which a single genetic variant or gene affects multiple phenotypic traits.
Polygenic Score (PGS): A single value estimate of an individual’s common genetic liability to a phenotype, calculated as the sum of risk alleles associated with a phenotype of interest, weighted by the corresponding effect size estimate from a GWAS.
Single Nucleotide Polymorphism (SNP): Common genetic variant at a single base pair in the genome that can vary between individuals.
SNP-based heritability: Proportion of variation in a trait or disease risk that is explained by the additive effects of common genetic variants (SNPs)
Whole Exome Sequencing (WES): Method used to identify statistically significant associations between protein-coding regions of genes (the exome) and a trait or disease within a population.
Whole Genome Sequencing (WGS): Sequencing method that determines the complete DNA sequence of an individual, including coding and non-coding regions.
